# *RET* fusions as primary oncogenic drivers and secondary acquired resistance to EGFR tyrosine kinase inhibitors in patients with non-small-cell lung cancer

**DOI:** 10.1186/s12967-022-03593-3

**Published:** 2022-09-04

**Authors:** Chunyue Wang, Zhenlong Zhang, Yulan Sun, Song Wang, Mengmeng Wu, Qiuxiang Ou, Yang Xu, Zhiming Chen, Yang Shao, Hong Liu, Peifeng Hou

**Affiliations:** 1 Department of Medical Oncology, Xiamen Key Laboratory of Antitumor Drug Transformation Research, The First Affiliated Hospital of Xiamen University, School of Medicine, Xiamen University, Xiamen, 361013 Fujian China; 2 Department of Thoracic Surgery, Shengli Clinical Medical College of Fujian Medical University, Fujian Provincial Hospital, Fuzhou, 350001 Fujian China; 3 Department of Internal Medicine Division, Shandong Cancer Hospital Affiliated to Shandong First Medical University, Jinan, 250117 Shandong China; 4 Geneseeq Research Institute, Nanjing Geneseeq Technology Inc., Nanjing, 210032 Jiangsu China; 5 Affiliated Hospital of Nantong University, Nantong, 226001 Jiangsu China; 6 Department of Radiation Oncology, Qilu Hospital of Shandong University, Jinan, 250012 Shandong China; 7 Department of Medical Oncology, Fujian Medical University Union Hospital, Fuzhou, 350001 Fujian China

**Keywords:** *RET* rearrangement, NSCLC, EGFR-TKI, *KIF5B*, *CCDC6*, Noncanonical *RET* fusion

## Abstract

**Background:**

*RET* fusions are rare oncogenic drivers in non-small cell lung cancer (NSCLC). While activating *RET* rearrangements are found in NSCLC patients harboring epidermal growth factor receptor (*EGFR*) genetic alterations at resistance to EGFR inhibitors, the extent to which co-occurring genomic alterations exist and how they might affect prognosis or therapy response is poorly understood.

**Methods:**

Targeted next-generation sequencing (NGS) was used to assess 380 baseline patients with primary *RET* fusions and 71 *EGFR*-mutated NSCLC patients who acquired *RET* fusions after developing resistance to EGFR-tyrosine kinase inhibitors (EGFR-TKIs).

**Results:**

Primary *RET* fusions were more likely associated with females and younger age, with *KIF5B* being the predominant fusion partner. In baseline patients, both *SMAD4* (5.3% vs. 0.0%, *P* = 0.044) and *MYC* copy-number gain variants (6.9% vs. 0.0%, *P* = 0.009) were more frequently co-mutated with *KIF5B-RET* than *CCDC6-RET*. By contrast, *CDKN2A* (11.3% vs. 2.4%, *P* = 0.003) mutations were significantly enriched in *CCDC6-RET*-rearranged baseline patients. A significant increase in the proportion of *CCDC6-RET* was observed in acquired *RET*-rearranged patients (47.3% vs. 22.5%, *P* < 0.001). The median progression-free survival (PFS) of patients harboring *RB1* and *TP53* double-mutations (5.5 vs. 10.0 months, *P* = 0.020) or *ERBB2* amplification (5.6 vs. 10.0 months, *P* = 0.041) was significantly shorter than the wild-type counterparts. Moreover, we identified that *RET* fusions were more likely associated with acquired resistance (AR) to third-generation EGFR-TKIs than previous generations of EGFR-TKIs.

**Conclusions:**

In conclusion, we depicted the mutational profiles of NSCLC patients who harbor *RET* fusions at baseline or after resistance to EGFR-TKIs. Furthermore, our results suggest that *RET* fusions mediate secondary resistance to third-generation EGFR-TKIs and might be associated with poor prognosis in patients with NSCLC.

**Supplementary Information:**

The online version contains supplementary material available at 10.1186/s12967-022-03593-3.

## Background

Non-small-cell lung cancer (NSCLC) is the leading cause of cancer-related mortality, which accounts for more than 80% of lung cancers, with lung adenocarcinoma (ADC) being the most common histological type. *RET* (*Rearranged during transfection*) gene fusions are present in approximately 1–2% of NSCLC and have emerged as a targetable oncogenic driver for NSCLC patients [1–4].

The fusion of *RET* with another unrelated gene occurs due to an aberrant DNA repair process [2]. The resulting fusion product activates various downstream signaling pathways that play essential roles in cell proliferation and survival [5]. Previous studies have shown that *RET* fusion-positive (*RET*+) NSCLC patients testing negative for *EGFR*/*ALK*/*BRAF*/*ROS1* are usually young never-smokers with ADC [6–9]. While the most common *RET* fusion partners in NSCLC patients are *KIF5B* and *CCDC6*, other reported partners include *NCOA4*, *TRIM33*, etc. [10]. The overall survival of *CCDC6-RET*+ baseline patients was nearly three times longer than those with *KIF5B-RET* fusions (median: 113.5 vs. 37.7 months, *P* = 0.009) [11]. Besides, treatment responses of *RET* inhibitors were heterogeneous among baseline *RET*+ patients harboring different fusion variants [9, 11–14], highlighting the importance of fusion partner types in clinical outcomes toward targeted therapy.

More recently, receptor tyrosine kinase (RTK) fusions have emerged as a rare but targetable acquired resistance (AR) mechanism in *EGFR*-mutated NSCLC patients on EGFR-TKI treatment. Notably, *RET* fusions are the most commonly reported RTK fusions that mediate AR to EGFR-TKIs [15]. Within secondary *RET* fusions, *CCDC6-RET* is the most common fusion variant, followed by *NCOA4-RET*. Interestingly, NSCLC patients harboring *KIF5B-RET* fusions showed minimal response after RET TKI (RXDX-105) treatment, whereas the response rate was 67% in non-*KIF5B-RET*+ NSCLC patients [16]. Dual blockade of *EGFR* driver mutation and *RET* fusion, such as *CCDC6-RET* and *NCOA4-RET*, demonstrated safety and clinical efficacy in both clinical and preclinical studies [17].

To date, two highly potent *RET*-specific TKIs, selpercatinib and pralsetinib, have been approved by the US Food and Drug Administration (FDA) for the treatment of advanced or metastatic *RET*-altered NSCLC and thyroid cancers. Selpercatinib and pralsetinib effectively against *RET* alterations, including *CCDC6-RET* and *KIF5B-RET* fusions, *RET* activating mutations (C634W and M918T), and *RET* gatekeeper mutations V804L/M/E [18, 19]. Remarkably, selpercatinib has > 100-times selectivity against VEGFR2, and pralsetinib has 87-times selectivity against VEGFR2 and 20-times selectivity against JAK1 [20]. Findings from the phase I/II LIBRETTO-001 trial (NCT03157128) demonstrated that selpercatinib has an overall response rate (ORR) of 64% in previously treated NSCLC patients and ORR of 85% in treatment-naïve *RET*-altered NSCLC patients [21]. In addition, the antitumor potential of selpercatinib is irrespective of specific *RET* fusion types. On the other hand, initial data from the phase I/II ARROW trial (NCT03037385) demonstrated that pralsetinib has a high potency and durable activity and is well-tolerated in adult patients with metastatic *RET*-altered NSCLC. The ORR in previously treated patients was 61%, and ORR in treatment-naïve patients was 70% [22]. Most treatment-related adverse events (TRAE) of selpercatinib and pralsetinib are mild and controllable, including anemia, elevated alanine aminotransferase and hypertension [5, 20]. However, the safety profiles of these two *RET* inhibitors need to be further studied, given that some safety warnings have been reported [20–24].

In this study, we delineated the mutational profiles of 380 baseline and 71 *EGFR*-mutated NSCLC patients who acquired *RET* fusions after resistance to EGFR-TKIs by targeted NGS and revealed *RET* fusion partners associated with primary and acquired patients. We also investigated the impact of co-occurring genetic alterations in *RET*-rearranged NSCLC patients, which might explain the poor prognosis of patients harboring secondary *RET* fusions.

## Methods

### Patients and sample collection

Tumor tissue and/or plasma samples were collected from 451 *RET*+ NSCLC patients admitted to all participating hospitals between June 2015 and June 2021. Specifically, formalin-fixed paraffin-embedded (FFPE) tumor or fresh tumor tissue were confirmed by pathologists from the centralized clinical testing center. 5–10 mL of peripheral blood was collected from each patient in EDTA-coated tubes (BD Biosciences) and shipped to the clinical testing center within 48 h of blood collection for the following tests. Clinical characteristics and treatment history were extracted from medical records. Progression-free survival (PFS) was defined as the time from the initiation of the treatment to disease progression/patient death. Patients who had not progressed were censored at the date of their last follow-up. This study was conducted in accordance with the declaration of Helsinki and was approved by the Ethical Review Board of Fujian Medical University Union Hospital. Informed written consent was obtained from each subject before sample collection.

### Targeted next-generation sequencing

DNA extraction, library construction, and targeted NGS were performed as previously described in a Clinical Laboratory Improvement Amendments (CLIA)-certified and College of American Pathologists (CAP)-accredited clinical testing laboratory (Nanjing Geneseeq Technology Inc., Nanjing, China) [25, 26]. FFPE samples were de-paraffinized with xylene followed by genomic DNA extraction using QIAamp DNA FFPE Tissue Kit (Qiagen Cat. No. 56404) according to the manufacturer’s instructions. Genomic DNA from fresh tumor tissue was extracted using the DNeasy Blood and Tissue Kit (Qiagen Cat. No. 69504) according to standard protocols. Peripheral blood samples were centrifuged at 1800*g* for 10 min, followed by cell-free DNA (cfDNA) extraction and purification using QIAamp Circulating Nucleic Acid Kit (Qiagen Cat. No. 55114). Genomic DNA of white blood cells in sediments was extracted using the DNeasy Blood and Tissue Kit (Qiagen Cat. No. 69504) as normal control. Genomic DNA was qualified using Nanodrop2000 (Thermo Fisher Scientific, Waltham, MA), and cfDNA fragment distribution was analyzed on a Bioanalyzer 2100 using the High Sensitivity DNA Kit (Agilent Technologies, Santa Clara, CA, 5067-4626). DNA quantification was performed using the dsDNA HS assay kit on a Qubit 3.0 fluorometer (Life Technology, US). NGS libraries were prepared using the KAPA Hyper Prep kit (KAPA Biosystems) with an optimized manufacturer’s protocol for different sample types. Targeted capture enrichment was performed as previously described [27]. The target-enriched library was then sequenced on HiSeq4000 or HiSeq4000 NGS platforms (Illumina) according to the manufacturer’s instructions.

### Mutation calling

Sequencing data were processed as previously described [25]. In brief, the data was first demultiplexed and subjected to FASTQ file quality control using Trimmomatic [28]. Low-quality data (QC below 15) and N bases were removed. Raw reads were then mapped to the Human Genome (hg19) using Burrows-Wheeler Aligner (BWA-mem, v0.7.12; https://github.com/lh3/bwa/tree/master/bwakit). Genome Analysis Toolkit (GETK 3.4.0; https://software.broadinstitute.org/gatk/) was employed to perform local realignment, base quality score recalibration, and detect germline mutations. Picard was used to remove PCR duplicates. VarScan2 was applied to detect single-nucleotide variations (SNVs) and insertion/deletion mutations. The limit of detection (LOD) of tumor tissues and plasma samples under specific sequencing depths has been tested repetitively in Nanjing Geneseeq Technology Inc. to ensure the mutation calling results are consistent and have optimal performance for identifying genetic alterations. According to its internal specifications, sequencing of tissue and plasma ctDNA via targeted NGS can reach a sensitivity of 98% and a positive predictive value (PPV) of 95% [26, 29, 30]. SNVs were filtered out if the VAF was less than 1% for tumor tissue and 0.3% for plasma samples. Common SNVs were excluded if they were present in > 1% population in the 1000 Genomes Project or the Exome Aggregation Consortium (ExAC) 65,000 exomes database. The resulting mutation list was further filtered by an in-house list of recurrent artifacts based on a normal pool of whole blood samples. Parallel sequencing of matched white blood cells from each patient was performed to remove sequencing artifacts, germline variants, and clonal hematopoiesis. Genomic fusions were identified by FACTERA [31] with default parameters (≥ 2 reads). The fusion reads were manually reviewed and confirmed on Integrative Genomics Viewer (IGV). By definition, *RET* fusions were annotated as a protein fusion involving: (i) a 5′ non-*RET* partner gene and (ii) an intact 3′ *RET* kinase domain (NM_020975: exon 12–18). In this study, *RET* fusions were classified into canonical (single or compound *KIF5B-RET* and *CCDC6-RET*) and noncanonical *RET*. In this regard, a noncanonical *RET* fusion includes: (i) a rearrangement with a partner rather than *KIF5B* and *CCDC6*; (ii) A rearrangement with a novel partner gene; and (iii) a rearrangement with an intergenic region (IGR). Tumor mutational burden (TMB, mutation per Megabase) was determined based on the number of missense mutations in the targeted regions of the gene panel covering 0.85 Mb of coding genome, excluding known driver mutations as they are over-represented in the panel. Chromosome instability score (CIS) was defined as the proportion of the genome with aberrant (purity-adjusted segment-level copy number ≥ 3 or ≤ 1) segmented copy number [32]. For gene-level analyses in the baseline cohort, genetic alterations with a mutation frequency ≥ 2% were considered frequently mutated. All 139 genes in the PULMOCAN™ gene panel (Geneseeq Technology Inc.) were subjected to pathway enrichment analyses. Specifically, the genetic alterations identified by targeted panel sequencing were first categorized into ten signaling pathways associated with common hallmarks of cancer that control cell-cycle progression, apoptosis, cell proliferation and growth [33]. The proportion of baseline patients with specific *RET* fusion variants harboring mutation(s) in the relevant pathways was compared to reveal the differences of co-existing mutations in baseline *RET*+ patients.

### Statistical analysis

Fisher’s exact tests were used to compare the categorical variables between groups. Kaplan–Meier curves were used to analyze the PFS of various patient groups, and the statistical difference was analyzed using the log-rank test. A two-sided *P* value of less than 0.05 was considered significant for all tests unless indicated otherwise (*P < 0.05, 0.01 < **P < 0.05, ***P < 0.001). All statistical analyses were performed using R (version 4.1.2).

## Results

### Patient overview

**Fig. 1:**
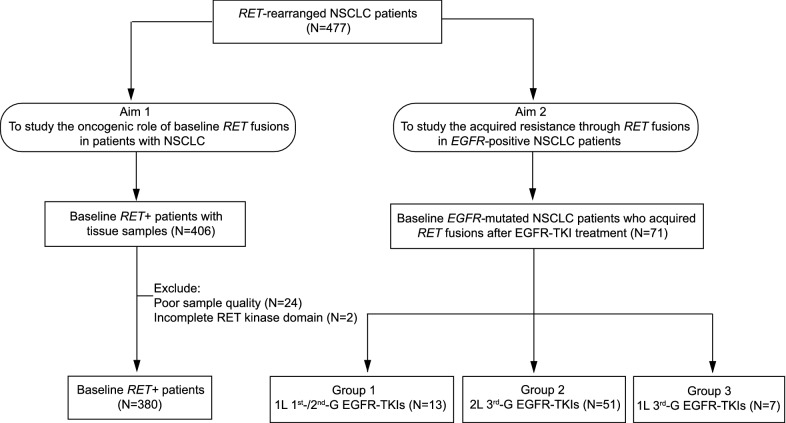
Patient overview. A total of 451 patients were included in the final analysis, of which 380 were baseline *RET*+ patients and 71 were *EGFR*-mutated patients who acquired *RET* fusions after resistance to EGFR-TKIs. Only baseline *RET* fusion-positive (*RET*+) patients with available FFPE and/or biopsy tumor samples were qualified for the following tests. Acquired *RET*+ patients (N = 71) were those who gained *RET* fusions after EGFR-TKI treatments targeting primary *EGFR* oncogenic mutations. Samples belonging to this category were divided into three groups depending on when *RET* fusion was detected. Specifically, group 1 contains 13 patients treated with first-line (1L) 1st- or 2nd-G EGFR-TKIs. Group 2 includes 51 patients who received second-line (2L) 3rd-G EGFR-TKI treatment. Lastly, group 3 consists of 7 patients previously treated with 1L 3rd-G EGFR-TKIs

After excluding patients with either poor NGS quality samples or incomplete RET kinase domain fusions, 451 patients with NSCLC harboring *RET* fusions were included in the study cohort (Fig. 1). In particular, 380 patients had baseline *RET* fusions in EGFR-TKI treatment-naïve tissue samples, whereas 71 patients who carried baseline *EGFR* mutations acquired *RET* fusions after resistance to EGFR-TKIs. According to the EGFR-TKI treatment regimens, the 71 acquired *RET*+ patients were subdivided into three groups: (i) patients treated with first-line (1L) 1st- or 2nd-generation (G) EGFR-TKIs (N = 13), (ii) patients treated with second-line (2L) 3^rd^-G EGFR-TKIs (N = 51), and (iii) patients treated with 1L 3^rd^-G EGFR-TKIs (N = 7).

In the baseline cohort, 58.9% of patients were under 60 years (58.9% vs. 41.1%, *P* < 0.001), and the proportion of females was significantly higher than males (55.3% vs. 44.7%, *P* = 0.038, Table 1). Of the 71 patients with acquired *RET* fusions, a higher proportion of younger (63.4% vs. 36.6%, *P* = 0.024) and female patients (64.8% vs. 35.2%, *P* = 0.013) was observed. In both baseline and acquired *RET*+ patient cohorts, ADC was the predominant histological subtype in NSCLC patients. All 71 patients harbored baseline *EGFR* mutations, including 47 with exon 19 deletions (19-Del, 66.2%), 23 with substitution mutation L858R (32.4%) and one G719C/S768I double-mutant patient (1.4%).

**Table 1:** Clinical characteristics of patients

	N (%)			*P* value*
	Total (N = 451)	Baseline (N = 380)	Acquired (N = 71)	
Age				0.5126
< 60	269 (59.6)	224 (58.9)	45 (63.4)	
≥ 60	182 (40.4)	156 (41.1)	26 (36.6)	
Gender				0.1522
Female	256 (56.8)	210 (55.3)	46 (64.8)	
Male	195 (43.2)	170 (44.7)	25 (35.2)	
Histology				8.37E-09
ADC	351 (77.8)	289 (76.1)	62 (87.3)	
ASC	6 (1.3)	4 (1.1)	2 (2.8)	
SCC	5 (1.1)	4 (1.1)	1 (1.4)	
LCC	1 (0.2)	0 (0.0)	1 (1.4)	
PSC	7 (1.6)	2 (0.5)	5 (7.0)	
NOS	81 (18.0)	81 (21.3)	0 (0.0)	
Stage at diagnosis				–
I	–	70 (18.4)	–	
II	–	6 (1.6)	–	
III	–	12 (3.2)	–	
IV	–	163 (42.9)	–	
Unknown	–	129 (33.9)	–	
*EGFR* mutation type				–
19-Del	–	–	47 (66.2)	
L858R	–	–	23 (32.4)	
G719C/S768I	–	–	1 (1.4)	
EGFR-TKI treatment				–
1L 1st/2nd-G EGFR-TKIs	–	–	13 (18.3)	
2L 3rd-G EGFR-TKIs	–	–	51 (71.8)	
1L 3rd-G EGFR-TKIs	–	–	7 (9.9)	

Statistical analysis between baseline and acquired patients was based on Fisher’s exact test. ADC, adenocarcinoma; ASC, adenosquamous carcinoma; SCC, squamous cell carcinoma; LCC, large cell carcinoma; PSC, pulmonary sarcomatoid carcinoma; NOS, not otherwise specified; 1L, first-line; 2L, second-line

### Distribution of *RET* fusion partners between primary and acquired *RET*+ patients

**Fig. 2:**
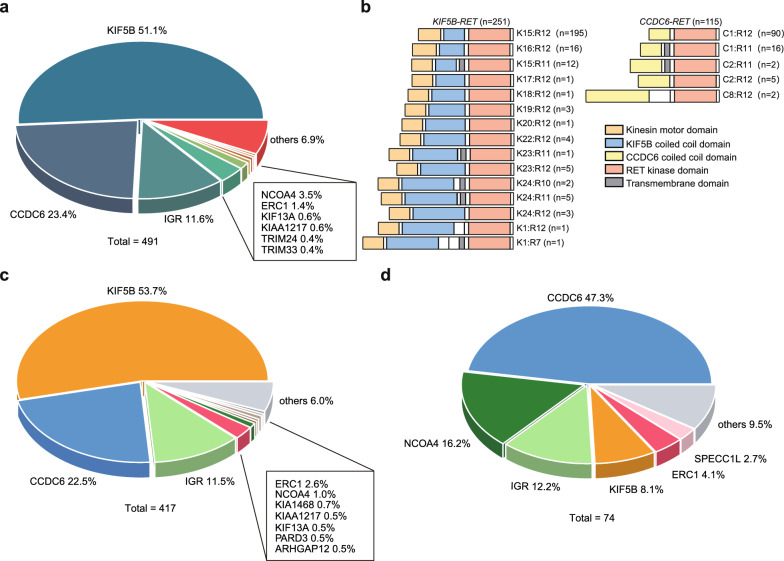
Distribution of *RET* fusions. **a** *RET* fusions identified by targeted NGS in the total patient cohort (N = 451). **b** A schematic demonstration of functional domains in *KIF5B*-*RET* and *CCDC6*-*RET* fusions identified in the study cohort. Descriptions on the right indicate exons in the partner gene and *RET* gene. KIF5B-RET fusion proteins contain the kinesin motor domain (orange), the coiled coil domain (blue) from KIF5B, and the kinase domain (pink) of RET, or the transmembrane domain (grey) and the kinase domain of RET. CCDC6-RET fusions contain the coiled coil domain (yellow) of CCDC6 and the kinase domain of RET, or the transmembrane domain and the kinase domain of RET. **c, d** Distribution of *RET* fusion partner genes in baseline (**c**) and acquired (**d**) *RET*+ patients

By targeted NGS, a total of 491 *RET* fusions were identified in 451 *RET*+ patients (Fig. 2a), of which *KIF5B*-*RET* (51.1%) was the most frequently observed *RET* rearrangement, followed by *CCDC6-RET* (23.4%). Notably, 39 patients (8.6%) carried more than one *RET* fusion, of whom 36 were baseline and 3 were acquired *RET*+ patients. Next, we tried to depict functional domains and breakpoints of canonical *RET* fusions. In particular, 78% of *KIF5B*-*RET* (195/251) fusions contained the kinesin motor domain and coiled coil domain from exons 1–15 of *KIF5B* and the kinase domain from exons 12–18 of *RET* (Fig. 2b). In comparison, 78% (90/115) of *CCDC6*-*RET* fusions contained a fused coiled coil domain from exon 1 of *CCDC6* and the kinase domain from exons 12–18 of *RET*. These results were consistent with previous findings, showing that most genomic breakpoints are located within intron 11 of *RET*, while translocation events may also occasionally occur within intron 7 and 10, resulting in the inclusion of the RET transmembrane domain [34].

The next question we wanted to address was whether the percentage of specific *RET* fusion variants differs in baseline and acquired *RET*+ patients. We identified 417 and 74 *RET* rearrangements in these two cohorts, respectively (Fig. 2c, d). The most commonly observed fusion variants in baseline patients were *KIF5B-RET* (53.7%), followed by *CCDC6-RET* (22.5%, Fig. 2c). Surprisingly, *KIF5B*-*RET* accounted for only 8.1% of total translocation events in baseline *EGFR*-mutated NSCLC patients resistant to EGFR-TKIs, whereas *CCDC6-RET* was identified in 47.3% of translocations events, ranking as the predominant *RET* fusion variant in acquired *RET*+ patients (Fig. 2d). The identification of *CCDC6-RET* was the most common fusion variant, followed by *NCOA4-RET*, was consistent with previous findings [15]. Furthermore, these results implied that *KIF5B-RET* and non-*KIF5B-RET* fusions might have different functionalities in NSCLC. At the same time, noncanonical *RET* fusions, including those with hitherto unreported partner genes [10], were identified in 23.8% and 44.6% of baseline and secondary patient cohorts, respectively (Additional file 1: Table S1).

As *RET* fusion variants distributed differentially in baseline and acquired *RET*+ patients, we investigated whether *RET* fusion types could be associated with the patient's clinical manifestations. Interestingly, females were more likely associated with *KIF5B*-*RET* (*P* = 0.006) and noncanonical *RET* fusions (*P* = 0.015) than *CCDC6*-*RET* in baseline patients (Additional file 2: Fig. S1a). However, neither the patient’s age nor cancer stage at diagnosis was directly associated with *RET* fusion types in baseline or secondary patients (Additional file 2: Fig. S1b-e).

### *RET* fusion as a primary oncogenic driver in NSCLC patients

**Fig. 3:**
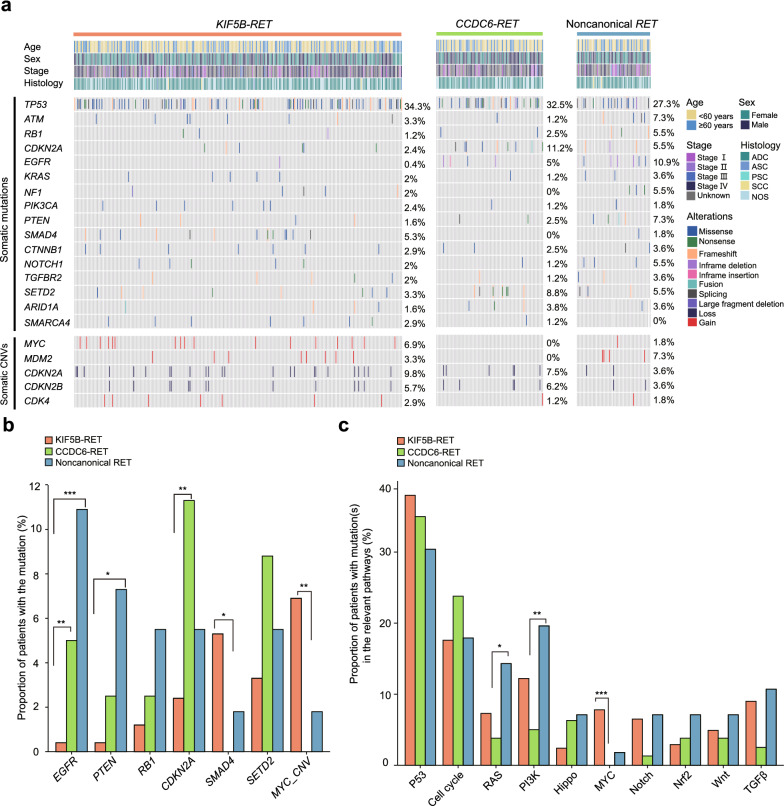
The genomic landscape of baseline *RET* fusion-positive patients. **a** Distribution of genetic alterations associated with baseline *RET*+ patients (N = 380). The distribution of somatic mutations (top) and CNVs (bottom) in baseline patients were assessed by targeted NGS. Each column represents one patient. Clinical characteristics of baseline *RET*+ patients are shown at the top. The frequency of each gene alteration is listed on the right. **b** Top frequently mutated gene alterations identified in *RET* + baseline patients. *P < 0.05, **P < 0.01, ***P < 0.001. **c** The correlation between signaling pathways in which the concurrent mutations occur and different types of *RET* fusions. The bar graph illustrates the proportion of baseline *RET* fusion-positive patients harboring genetic alterations in the relevant pathways. *P < 0.05, **P < 0.01, ***P < 0.001

*RET* fusions are oncogenic drivers that usually occur mutually exclusive to other driver genes in NSCLC patients. Research to date has not yet addressed why *RET*-rearranged patients with different fusion partners responded differently to treatments. We hypothesized that this could result from different mutational profiles among baseline *RET*+ patients. Accordingly, we analyzed the NGS data of 380 baseline *RET*+ patients and profiled their genomic landscape. Genetic alterations, such as *TP53*, *MDM2*, *CDKN2A*/*B*, *ATM*, and *RB1*, were frequently detected in baseline *RET*+ patients (Fig. 3a). Notably, although 94.5% (360/380) of our baseline *RET*+ patients contained only *RET* fusions as the primary oncogenic driver of NSCLC, 21 patients (5.5%) harbored additional driver gene aberrations, including *EGFR* (11/21), *KRAS* (6/21), *BRAF* (2/21), *ERBB2* (1/21) and *ROS1* (1/21) (Additional file 3: Fig. S2a). However, there was no significant correlation between the patient number and the patient’s clinical characteristics (Additional file 3: Fig. S2b).

Our next objective was to investigate whether specific genomic alterations could be associated with baseline patients harboring specific *RET* fusion variants. Hence, we performed both gene- and pathway-level enrichment analyses using targeted NGS results of tumor tissues collected from 380 baseline *RET*+ patients. *EGFR* mutations were more commonly found in patients with *CCDC6-RET* (5.0% vs. 0.4%, *P* = 0.014) and noncanonical *RET* fusions (7.1% vs. 0.4%, *P* < 0.001) compared to *KIF5B-RET* fusions (Fig. 3b). Meanwhile, co-existing mutations in *PTEN* were more frequently associated with noncanonical *RET* fusions than *KIF5B-RET* fusions (7.1% vs. 0.4%, *P* = 0.042). In addition, *CDKN2A* mutations were more frequently co-existed with *CCDC6-RET* than *KIF5B-RET* (11.3% vs. 2.4%, *P* = 0.003), while *SMAD4* mutations (5.3% vs. 0.0%, *P* = 0.044) and *MYC* amplification (6.9% vs. 0.0%, *P* = 0.009) were more frequently found in patients with *KIF5B*-*RET* fusions than those with *CCDC6-RET* fusions. By performing pathway-level analyses, we noticed that noncanonical *RET* fusions were more likely to be associated with mutations in the PI3K and RAS/RTK pathways (Fig. 3c). In great contrast, the aberrant MYC pathway more frequently co-occurred with *KIF5B-RET* than *CCDC6-RET* (7.8% vs. 0.0%, *P* < 0.001) in baseline *RET*-rearranged patients with NSCLC. None of the other oncogenic pathways examined showed a significant difference among patients harboring *KIF5B-RET* or *CCDC6-RET* fusions.

Lastly, we examined tumor mutational burden (TMB) and chromosomal instability in baseline *RET*+ patients, where we found no significant difference in TMB, chromosomal instability score (CIS), or arm-level copy number variations (CNVs) among different types of *RET* fusions (Additional file 4: Fig. S3). From the above analysis, we depicted mutational profiles of *RET*+ patients and identified concomitant mutations that may contribute to the differential treatment responses in *RET-*rearranged patients.

### *RET* fusion confers a secondary resistance mechanism to EGFR-TKIs

**Fig. 4:**
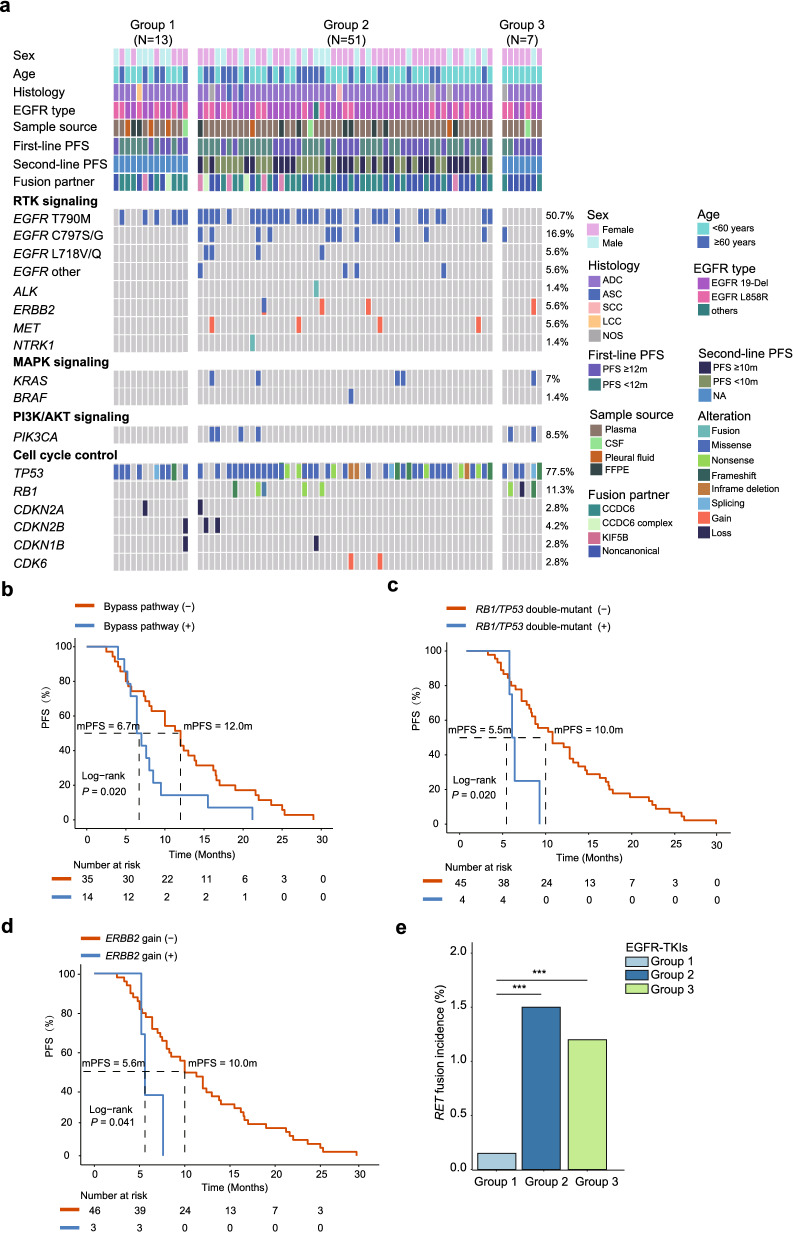
Somatic gene alterations identified in acquired *RET*+ patients who exhibit resistance to EGFR-TKIs. **a** Genomic landscape of somatic gene alterations in patients with acquired *RET* fusions (N = 71). Grouping of patients is based on EGFR-TKI treatment regimens. Each column represents one patient. The clinical characteristics of each patient are shown on the top. The percentage on the right shows the mutation frequency of each gene. Grouping of second-line PFS was in line with the results of the AURA3 study (mPFS duration of 10.1 months) [43]. **b** Kaplan–Meier estimates of PFS in acquired *RET*+ patients who previously received 2L 3^rd^-G EGFR-TKI therapy (N = 49, see Additional file 5: Fig. S4b for patient assortment) with or without bypass pathway mutations (activating mutations in *KRAS* and *PIK3CA*, copy-number gain in *ERBB2* and *MET*, fusions in *ALK* and *NTRK*). **c** Kaplan–Meier estimates of PFS comparing patients with or without double-mutated *RB1* and *TP53* genes. **d** Kaplan–Meier estimates of PFS comparing patients with or without *ERBB2* amplification. **e** The incidence of *RET* fusions in *EGFR-*mutant NSCLC patients previously treated with different EGFR-TKIs

Despite their rarity, it is clear from previous studies that *RTK* fusions, such as *RET* rearrangements, are actionable resistance mechanisms to EGFR-TKIs. We aim to increase awareness of this emerging paradigm by comprehensively profiling baseline *EGFR*-mutated NSCLC patients who acquired *RET* fusions after developing resistance to EGFR-TKIs. In acquired *RET*+ patients, second-site *EGFR* mutations, such as T790M (50.7%), C797S/G (16.9%) and L718V/Q (5.6%), were among the top frequently mutated gene alterations (Fig. 4a). It was also apparent that cell cycle control pathway gene alterations, such as *TP53*, *RB1*, *CDKN2A*, *CDKN2B*, *CDKN1B*, and *CDK6*, co-existed with acquired *RET* fusions. In addition, *ALK* and *NTRK1* fusions that belong to the RTK family were identified to co-occur with *RET* fusion in two patients. These results implied that RTK fusions and other genetic variants could potentially serve as resistance mechanisms to anti-EGFR treatment in NSCLC.

To characterize the AR mechanism through *RET* fusions in *EGFR*-mutated NSCLC patients and their survival outcomes, we compared the PFS among patients treated with different EGFR-TKI regimens. However, no significant difference was observed (Additional file 5: Fig. S4a). Alternatively, as 2L 3rd-G EGFR-TKI treatment has been employed as a standard practice for *EGFR*-mutated NSCLC patients with progressive disease on first-line targeted therapy, we performed integrated survival analyses using group 2 patients. Notably, we excluded one patient with the rare *EGFR* double-mutants and one with SCC histology, leaving 49 patients with only *EGFR* 19-Del or L858R mutations in the refined cohort (Additional file 5: Fig. S4b). We first performed a univariate analysis on various demographic and mutational features of refined cohort 2 patients. Interestingly, bypass pathway genetic alterations (*KRAS* and *PIK3CA* activation mutations, *ERBB2* and *MET* amplification, *ALK* and *NTRK* fusions), *RB1* and *TP53* co-mutation, and *ERBB2* copy-number gain had significant associations with the prognosis of acquired *RET*+ patients who underwent 2L 3^rd^-G EGFR-TKI treatment (Additional file 1: Table S2). We then performed multivariate analysis using these three features, and we discovered that they were no longer statistically significant, with bypass mutations and *RB1*and *TP53* co-mutations being close to significance. This result suggested that there might be some interactions or correlations among these prognostic features. Nonetheless, the PFS of patients harboring bypass pathway gene alterations was significantly shorter than wild-type patients (median: 6.7 vs. 12.0 months, *P* = 0.020, Fig. 4b). In addition, the PFS of patients with *TP53* and *RB1* co-mutations was significantly shorter than wild-type counterparts (median: 5.5 vs. 10.0 months, *P* = 0.02, Fig. 4c). *ERBB2* gene amplifications that were present in 5.6% of acquired *RET*+ patients were also associated with poor prognosis in NSCLC patients (median: 5.6 vs. 10.0 months, *P* = 0.041, Fig. 4d). Notably, although the majority of acquired *RET* fusions were detected by circulating nucleic acid method using plasma ctDNA, 4 out of 49 patients in the refined cohort contributed only FFPE samples, raising a concern of missing rare fusions compared to tumor-based comprehensive genomic profiling. We, thereby, excluded patients with only tissue samples and re-performed the analysis in Fig. 4 using only ctDNA data (Additional file 6: Fig. S5a). Overall, our results about the co-occurring genetic alterations were consistent when using either all samples (tissue and ctDNA) or only ctDNA (Additional file 6: Fig. S5b-d), suggesting that our conclusions are not likely to be affected by different sample types.

Collectively, comprehensive molecular profiling of acquired *RET* + patients showed that co-existing genomic alterations, such as *TP53* and *RB1* co-mutations and *ERBB2* amplification, might be associated with a poor prognosis in *EGFR*-mutated NSCLC patients upon drug resistance.

### *RET* fusions are more frequently associated with third-generation EGFR-TKIs

Previous case reports have linked *RET* fusions, such as *CCDC6-RET*, *TRIM24-RET*, *NCOA4*-*RET* and *ERC1-RET*, to osimertinib resistance in *EGFR*-mutated NSCLC patients [35–39]. Here, we demonstrated a higher incidence rate of *RET* fusions in patients who developed AR to 3rd-G EGFR-TKIs from a population perspective. Interestingly, the incidence of *RET* fusions was highest in patients who underwent 2L 3rd-G EGFR-TKI treatment (1.5%, 51/3330), followed by patients treated with 1L 3rd-G EGFR-TKIs (1.2%, 7/589, Fig. 4e). These numbers were about ten-fold higher than those in patients treated with first- or second-generation EGFR-TKIs at the front line (0.15%, 13/8732). Overall, we demonstrated that *RET* fusions were more likely associated with *EGFR-*mutant NSCLC patients who received therapeutic interventions targeting EGFR with third-generation EGFR-TKIs. The complexity of non-*RET* secondary mutations in acquired *RET*+ patients might contribute to the differential treatment responses to follow-up therapies.

## Discussion

With comprehensive genomic profiling, clinicians can use the knowledge of specific clinical features associated with individual alterations to optimize therapeutic decision-making. Here, we retrospectively analyzed the mutational profiles of 451 patients carrying either baseline or acquired *RET* fusions to characterize the roles of *RET* fusions in NSCLC.

As a rare oncogenic driver mutation, *RET* rearrangement occurs in 1–2% of NSCLC patients [1, 2]. It has been shown that *RET*-rearranged NSCLC patients are more likely associated with ADC. Consistent with this notion, we found that 76.1% of patients developed ADC as the predominant histological type in the baseline cohort. On the other hand, a much-debated question is whether baseline *RET* fusions can correlate with the patient’s gender or age [8, 40, 41]. Hence, we compared clinical characteristics of baseline patients with *RET* fusions, such as gender and age. Our results showed that primary *RET* fusions were more likely to occur in females than males (55.3% vs. 44.7%, *P* = 0.038) and were associated with younger patients (58.9% vs. 41.1%, *P* < 0.001). The controversy could be due to variations in the subject’s ethnicity, genetic background, environmental factors, and even lifestyles. Then, we compared whether the distribution of specific *RET* fusions differed in baseline and acquired *RET*+ patients. We found that the predominant fusion type in baseline patients was *KIF5B-RET*, whereas *CCDC6-RET* was most frequently identified in patients who acquired *RET* fusions at resistance to EGFR-TKIs. The proportion of patients harboring *NCOA4-RET* fusions also increased from 1.0 to 16.2% in acquired *RET*+ patients. These results were consistent with previous findings, suggesting that *KIF5B-RET* and non-*KIF5B-RET* fusions might have different functionalities in EGFR-TKI progression [17, 35]. Lastly, we characterized mutational profiles of the 380 baseline patients and demonstrated that concurrent gene alterations, such as *SMAD4* mutations and *MYC* copy-number gain, were more frequently associated with *KIF5B-RET* than *CCDC6-RET* fusions in baseline *RET*-rearranged NSCLC patients. Our results may provide insights into why NSCLC patients harboring *KIF5B-RET* fusions have a nearly three times shorter overall survival than those harboring *CCDC6-RET* fusions.

The diversity and complexity of molecular mechanisms underlying the acquired adaptation of cancer cells to targeted therapies, such as EGFR-TKIs, is an area of active investigation. In this study, we demonstrated that *RET* fusions, as a rare but actionable AR mechanism to EGFR-TKIs, confer a poor prognosis in *EGFR*-mutated NSCLC patients. It has been previously suggested that patients harboring *TP53* and *RB1* co-mutations are at unique risk of histologic transition from ADC to SCC or eventually small cell transformation [42]. Here, our results showed that *RET*+ patients with co-occurring *TP53* and *RB1* double-mutations had significantly shorter PFS than wild-type patients, highlighting the role of *TP53* and *RB1* in controlling cell proliferation and disease progression. In addition, *ERBB2* copy-number gain can also present a similar effect, resulting in reduced PFS in patients who acquired *RET* fusions at resistance to EGFR-TKIs. It should also be noted that prognostic-related factors being examined in the survival analyses, including bypass pathway gene alterations, *TP53* and *RB1* co-mutations, and *ERBB2* copy-number gain, were not completely independent. The limited sample size may have impaired the statistical power to achieve significant results. Further studies using larger cohorts that consider additional variables need to be undertaken to better understand prognostic factors associated with the patient's survival.

In the final part of our study, we compared the incidence of secondary *RET* fusions in NSCLC patients who progressed on different EGFR-TKI therapies. A ten-fold higher incidence of *RET* fusions was observed in patients who underwent third-generation EGFR-TKI treatment than those treated with front-line 1st-/2nd-G EGFR-TKIs. This result was consistent with a previous finding which suggested that *RET* fusions were significantly enriched after 3rd-G EGFR-TKI treatment [15]. As previously mentioned, selpercatinib and pralsetinib have been granted FDA approval, showing equipotent for treating *RET*-rearranged NSCLC and thyroid cancer with minor and controllable adverse effects. The ORR of previously treated *RET*+ NSCLC patients on selpercatinib and pralsetinib were 64% and 61%, respectively, while the median PFS of previously treated patients on these two *RET* inhibitors was 16.5 months and 17.1 months, respectively [21, 22]. Given the high efficacy and mild side effects of the two *RET*-specific inhibitors, it is worth investigating their clinical utility to treat *EGFR*-mutated NSCLC patients who acquired *RET* oncogenic alterations after TKI resistance, especially those receiving 3rd-G EGFR-TKIs.

Despite efforts we made to systemically characterize the mutational profiles of baseline and acquired *RET*+ patients, there are limitations in our study. We did not validate the authenticity of *RET* fusions with hitherto unreported partner genes. However, we reasoned that patients without known driver mutations might harbor functional *RET* fusions since *RET* fusions usually occur mutually exclusively. Due to the unavailability of *RET*-specific inhibitors during the patient’s treatment, we could not evaluate the PFS of baseline patients with different *RET* fusion partners on *RET*-specific inhibitors or patients with acquired *RET* fusions after EGFR-TKI resistance. However, dynamic monitoring of these acquired *RET*+ patients is currently ongoing. We intend to investigate the treatment outcomes for *EGFR*-mutated NSCLC patients harboring acquired *RET* fusions on the follow-up *RET*-specific inhibitor therapy upon obtaining more data on these patients.

## Conclusions

In conclusion, we systematically evaluated the mutational profiles of *RET*+ baseline patients and those who acquired *RET* fusions at secondary resistance to EGFR-TKIs. We identified unique genetic features explaining the differential treatment responses in NSCLC patients harboring baseline *RET* fusions. Furthermore, we demonstrated that 3rd-G EGFR-TKIs are more likely associated with secondary *RET* fusions. The high efficacy and mild side effects of the two *RET*-specific inhibitors may provide treatment options for *EGFR*-mutated NSCLC patients who developed *RET* fusions following TKI resistance, especially those on 3rd-G EGFR-TKI treatments.

## Supplementary Information

**Additional file 1: Table S1.** Novel *RET* partner genes in NSCLC identified in the study cohort. **Table S2.** Univariate and multivariate analysis using the refined cohort.

**Additional file 2: Figure S1.** Correlation between patient’s clinical characteristics and *RET* fusion types. **a-c** Stacked bar plots demonstrate whether gender (a), age (b), and cancer stage at diagnosis (c) are associated with *RET* fusion subtypes in baseline *RET* + patients. Asterisks represent the significance level between two categorical variants based on Fisher’s exact test. **P < 0.01. **d-e** In acquired *RET* + patients, no significant correlation was observed between the patient’s gender (d) or age (e) and *RET* fusion types.

**Additional file 3: Figure S2.** Baseline *RET* fusions co-occurred with other oncogenic drivers. **a** The bar plot illustrates the percentage of baseline patients with (dark blue, 21/380) or without (yellow, 360/380) non-*RET* oncogenic driver mutations. The pie chart demonstrates the distribution of concurrent driver mutations in these patients. **b** Clinical characteristics of acquired *RET* + patients who harbored non-*RET* driver mutations.

**Additional file 4: Figure S3.** *RET* fusion type has no significant impact on TMB, CIS, or arm-level changes. No significant difference in TMB (a), chromosomal instability score (b) or arm-level changes (c-d) was observed among baseline patients with different *RET* fusions.

**Additional file 5: Figure S4.** The refinement of group 2 patients. **a** Kaplan–Meier estimates of PFS in patients treated with different EGFR-TKI regimens. **b** The flowchart demonstrates selecting qualified patients within group 2 for the following survival analyses. Patients with non-classic *EGFR* mutations or non-ADC histology were excluded.

**Additional file 6: Figure S5.** Survival analyses of patients with plasma ctDNA. **a** Patient stratification. Four patients with only FFPE samples were excluded from the refined cohort. **b-d** Kaplan–Meier estimates of PFS in patients with bypass pathway genetic alterations (b), *RB1* and *TP53* double-mutations (c), and *ERBB2* copy-number gain (c) versus corresponding wild-type patients.

## Data Availability

As the study involved human participants, the data cannot be made freely available in the manuscript nor a public repository because of ethical restrictions. However, the datasets generated and/or analyzed during this current study are available from the corresponding author on reasonable request.

## Abbreviations

NSCLC: Non-small cell lung cancer
*RET*: *Rearranged during transfection*
*RET*+: *RET* fusion-positive
*EGFR*: *epidermal growth factor*
EGFR-TKIs: EGFR-tyrosine kinase inhibitors
FDA: Food and Drug Administration
TRAE: Treatment-related adverse effects
G: Generation
NGS: Next-generation sequencing
PFS: Progression-free survival
AR: Acquired resistance
CLIA: Clinical Laboratory Improvement Amendments
CAP: College of American Pathologists
ADC: Adenocarcinoma
ASC: Adenosquamous cell carcinoma
SCC: Squamous cell carcinoma
LCC: Large cell carcinoma
NOS: Not otherwise specified
FFPE: Formalin-fixed paraffin-embedded
SNVs: Single-nucleotide variations
VAF: Variant allele frequency
IGR: Intergenic region
TMB: Tumor mutational burden
CIS: Chromosome instability score
19-Del: *EGFR* Exon 19 deletions
IGV: Integrative genomics viewer
1L: First-line
2L: Second-line
CNVs: Copy-number variations
HR: Hazard ratio
CI: Confidence interval
