# Supplementary material for: RET fusions as primary oncogenic drivers and secondary acquired resistance to EGFR tyrosine kinase inhibitors in patients with non-small-cell lung cancer
Source: J Transl Med. 2022 Sep 4;20:390. doi: 10.1186/s12967-022-03593-3 (PMC9441062; doi:10.1186/s12967-022-03593-3)
Supplement: Supplementary file 1 — Additional file 1: Table S1. Novel RET partner genes in NSCLC identified in the study cohort. Table S2. Univariate and multivariate analysis using the refined cohort. [file 12967_2022_3593_MOESM1_ESM.docx]

| **Supplementary Table S1. Novel *RET* partner genes in NSCLC identified in the study cohort** | | | |
| --- | --- | --- | --- |
| Sample type | Fusion partner | RET fusion | Additional driver mutations |
| Baseline | *c7orf61* | *C7orf61~RET* (int2:int11) | ~ |
|  | *CAMK1D* | *RET~CAMK1D* (int11:int5)  *KIF5B~RET* (int15:int11) | ~ |
|  | *CELF2* | *RET~CELF2* (int11:int1)  *KIF5B~RET* (int15:int11) | ~ |
|  | *FGF1* | *RET~FGF1* (int8:5’ UTR)  *KIF5B~RET* (int24:int9) | ~ |
|  | *GOLGA5* | *GOLGA5~RET* (int7:int11) | *ROS1* fusion |
|  | *NEURL1* | *RET~NEURL1* (exo11:int1)  *TRIM33~RET* (int11:exo11) | ~ |
|  | *RASGEF1A* | *RASGEF1A~RET*  (5’ UTR:exo11) | ~ |
|  |  | *RASGEF1A~RET* (5’ UTR:int11) | ~ |
|  | *SGMS1* | *SGMS1~RET* (5’ UTR:int11) | *EGFR* missense |
|  | IGR | n=38 | ~ |
|  | *AFF2* | *AFF2~RET* (int7:exo11) | ~ |
|  | *C10orf11* | *C10orf11~RET* (int5:int11) | ~ |
|  | *FXYD4* | *FXYD4~RET* (5’ UTR:int 11) | *KRAS* G13D |
|  | *RABEP1* | *RABEP1~RET* (int11:int1) | ~ |
|  | *TNIP1* | *TNIP1~RET* (int8:int11) | *BRAF* K601E |
|  | *VSTM4* | *VSTM4~RET* (int5:int11) | ~ |
| Acquired | *CDH23* | *CDH23~RET* (exo38:exo12) | ~ |
|  | *RIC1* | *RIC1~RET* (int2:exo11) |  |
|  | *SPECC1L* | *SPECC1L~RET* (exo14:exo12) | |
|  | IGR | n=8 |  |
|  | *RABEP2* | *RABEP2~RET* (int12:int11) | *FAM118A-NTRK1* fusion |

| **Supplementary Table S2. Univariate and multivariate analysis using the refined cohort** | | | | |
| --- | --- | --- | --- | --- |
|  | Univariate analysis | | Multivariate analysis | |
| Mutation | HR (95% CI) | *P* value | HR (95% CI) | *P* value |
| Age (<60 years) | 1.269 (0.439-1.416) | 0.426 | ~ | ~ |
| Gender (male) | 1.17 (0.637-2.15) | 0.613 | ~ | ~ |
| Bypass alterations | 2.16 (1.12~4.18) | **0.02** | 1.8 (0.87-3.72) | 0.115 |
| *RB1* and *TP53* co-mutation | 3.37 (1.13~10) | **0.02** | 2.28 (0.72-7.25) | 0.162 |
| *ERBB2* copy-number gain | 3.33 (0.96~11.5) | **0.0436** | 1.79 (0.47-6.88) | 0.395 |
| *EGFR* 19-Del | 0.79 (0.414~1.51) | 0.475 | ~ | ~ |
| *EGFR* L858R | 1.36 (0.72~2.56) | 0.343 | ~ | ~ |
| *EGFR* T790M | 0.847 (0.469~1.53) | 0.582 | ~ | ~ |
| *EGFR* others | 0.862 (0.453~1.64) | 0.651 | ~ | ~ |
| *TP53* | 1.16 (0.554~2.43) | 0.694 | ~ | ~ |
| *RB1* | 2.02 (0.84~4.85) | 0.11 | ~ | ~ |
| *KRAS* | 1.07 (0.382~3.01) | 0.895 | ~ | ~ |
| *PIK3CA* | 1.16 (0.489~2.75) | 0.736 | ~ | ~ |
| *CTNNB1* | 0.665 (0.281~1.57) | 0.35 | ~ | ~ |
| *SMAD4* | 0.973 (0.296~3.2) | 0.964 | ~ | ~ |
| *LRP1B* | 1.2 (0.427~3.38) | 0.727 | ~ | ~ |
| *ASXL1* | 0.825 (0.253~2.69) | 0.749 | ~ | ~ |
| *ARID1A* | 1.01 (0.358~2.82) | 0.991 | ~ | ~ |
| *DNMT3A* | 1.27 (0.522~3.1) | 0.596 | ~ | ~ |
| *KMT2A* | 1.73 (0.611~4.9) | 0.296 | ~ | ~ |
| *NKX-1* copy-number gain | 0.995 (0.306~3.23) | 0.993 | ~ | ~ |
| Bold represents significant *P* values based on the log-rank test. The Cox proportional hazard model was used for multivariate survival analysis. | | | | |
