# Supplementary figures and images for: RET fusions as primary oncogenic drivers and secondary acquired resistance to EGFR tyrosine kinase inhibitors in patients with non-small-cell lung cancer
Source: J Transl Med. 2022 Sep 4;20:390. doi: 10.1186/s12967-022-03593-3 (PMC9441062; doi:10.1186/s12967-022-03593-3)

**a**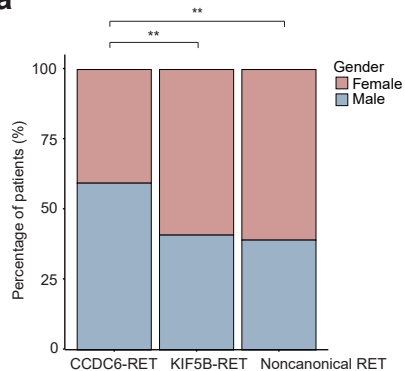**b**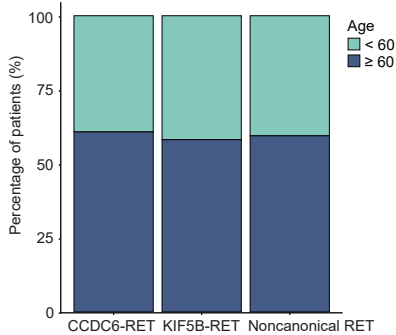**c**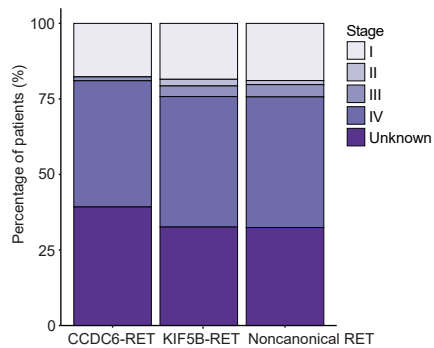**d**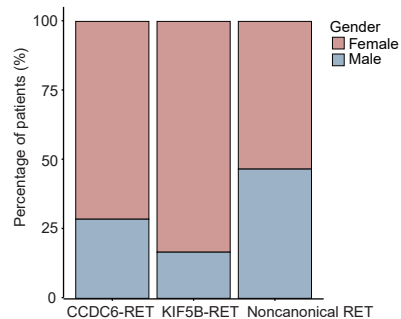**e**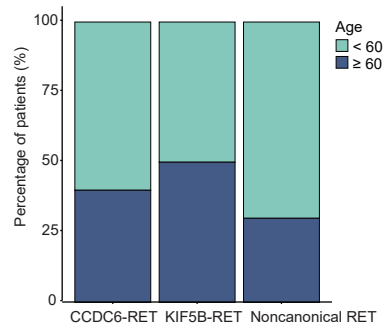

Supplement: Supplementary file 2 — Additional file 2: Figure S1. Correlation between patient’s clinical characteristics and RET fusion types. a-c Stacked bar plots demonstrate whether gender (a), age (b), and cancer stage at diagnosis (c) are associated with RET fusion subtypes in baseline RET + patients. Asterisks represent the significance level between two categorical variants based on Fisher’s exact test. **P < 0.01. d-e In acquired RET + patients, no significant correlation was observed between the patient’s gender (d) or age (e) and RET fusion types. [file 12967_2022_3593_MOESM2_ESM.pdf]

**a**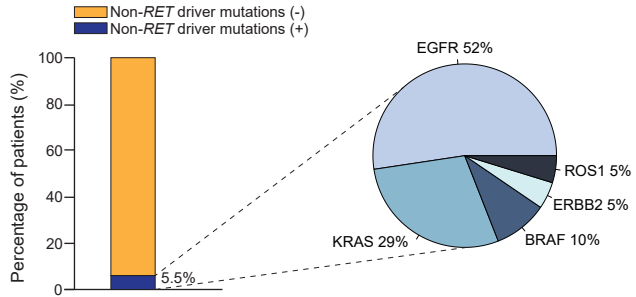**b**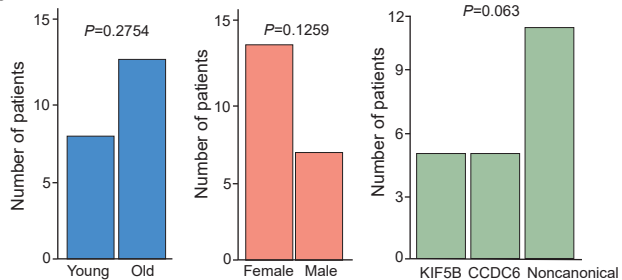

Supplement: Supplementary file 3 — Additional file 3: Figure S2. Baseline RET fusions co-occurred with other oncogenic drivers. a The bar plot illustrates the percentage of baseline patients with (dark blue, 21/380) or without (yellow, 360/380) non-RET oncogenic driver mutations. The pie chart demonstrates the distribution of concurrent driver mutations in these patients. b Clinical characteristics of acquired RET + patients who harbored non-RET driver mutations. [file 12967_2022_3593_MOESM3_ESM.pdf]

**a**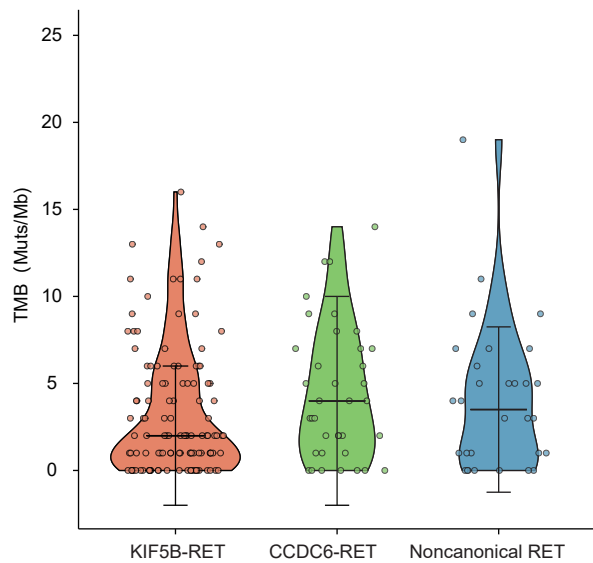**b**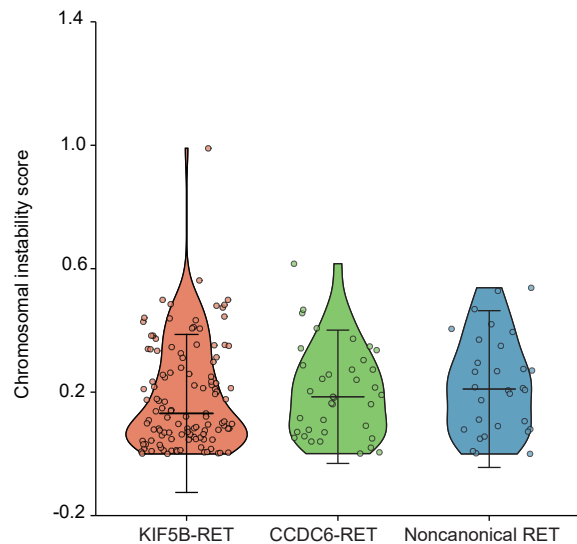**c**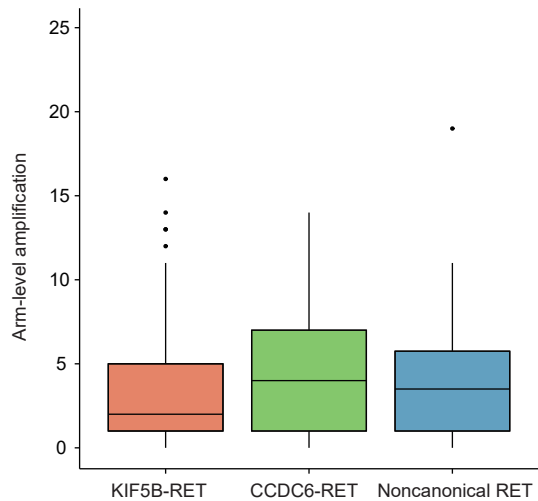**d**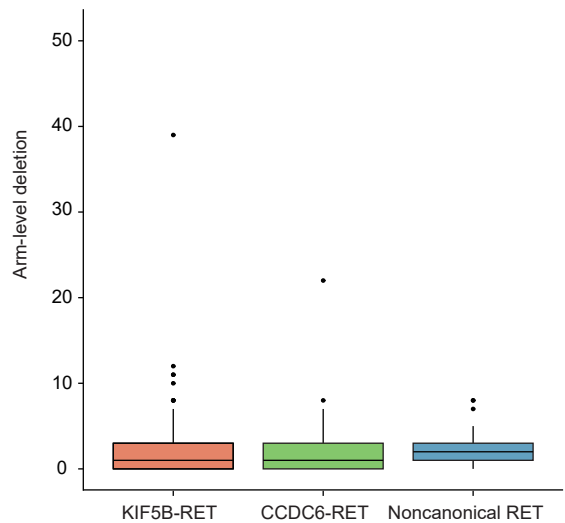

Supplement: Supplementary file 4 — Additional file 4: Figure S3. RET fusion type has no significant impact on TMB, CIS, or arm-level changes. No significant difference in TMB (a), chromosomal instability score (b) or arm-level changes (c-d) was observed among baseline patients with different RET fusions. [file 12967_2022_3593_MOESM4_ESM.pdf]

**a**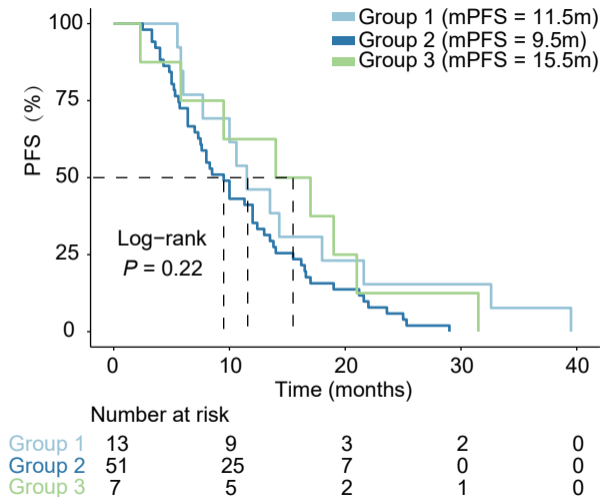**b**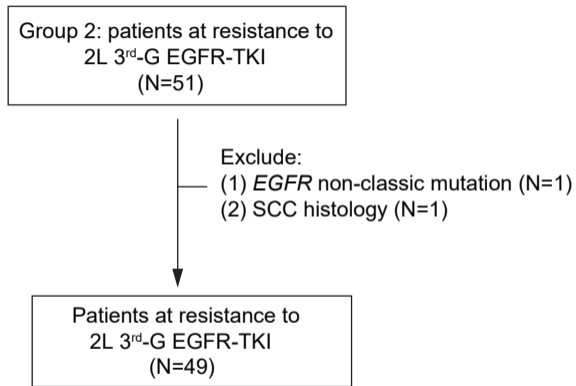

Supplement: Supplementary file 5 — Additional file 5: Figure S4. The refinement of group 2 patients. a Kaplan–Meier estimates of PFS in patients treated with different EGFR-TKI regimens. b The flowchart demonstrates selecting qualified patients within group 2 for the following survival analyses. Patients with non-classic EGFR mutations or non-ADC histology were excluded. [file 12967_2022_3593_MOESM5_ESM.pdf]

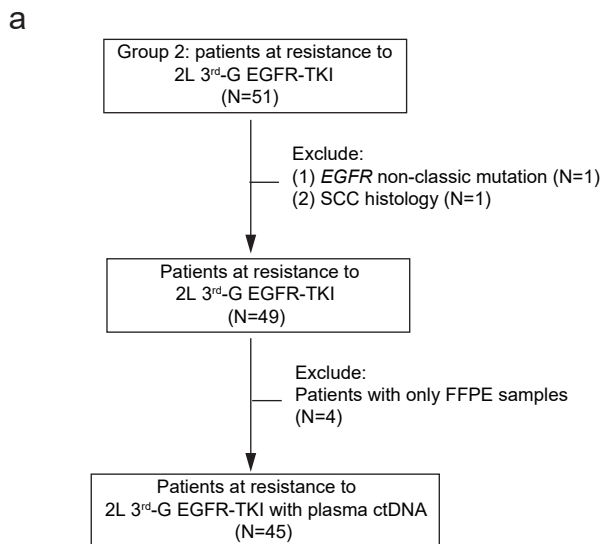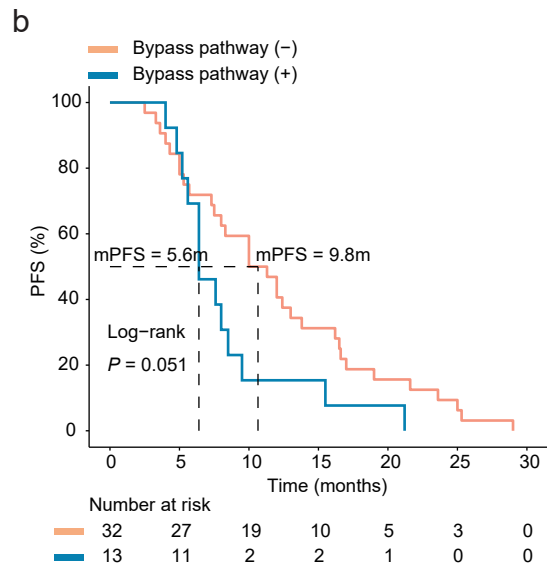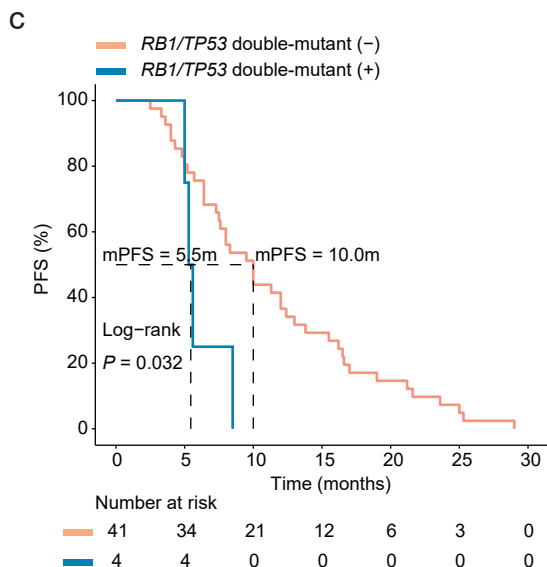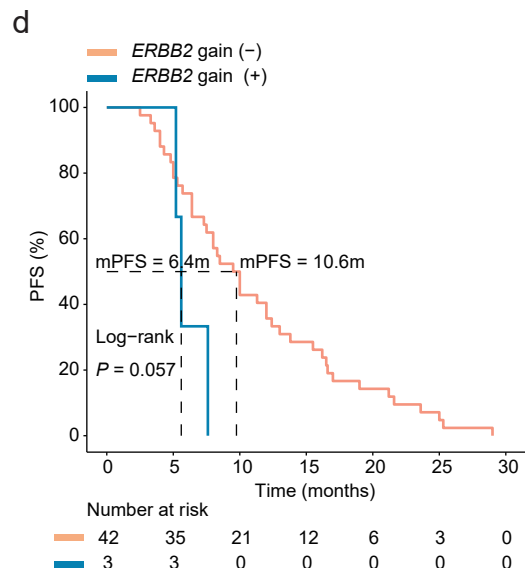

Supplement: Supplementary file 6 — Additional file 6: Figure S5. Survival analyses of patients with plasma ctDNA. a Patient stratification. Four patients with only FFPE samples were excluded from the refined cohort. b-d Kaplan–Meier estimates of PFS in patients with bypass pathway genetic alterations (b), RB1 and TP53 double-mutations (c), and ERBB2 copy-number gain (c) versus corresponding wild-type patients. [file 12967_2022_3593_MOESM6_ESM.pdf]
